# Validation of peripheral arterial tonometry as tool for sleep assessment in chronic obstructive pulmonary disease

**DOI:** 10.1038/s41598-019-55958-2

**Published:** 2019-12-18

**Authors:** Nils Henrik Holmedahl, Odd-Magne Fjeldstad, Harald Engan, Ingvild West Saxvig, Janne Grønli

**Affiliations:** 1 LHL Hospital Gardermoen, Jessheim, Norway; 2 Haukeland university hospital, Bergen, Norway; 3 University of Bergen, Bergen, Norway

**Keywords:** Respiration, Outcomes research, Biomedical engineering, Software

## Abstract

Obstructive sleep apnea (OSA) worsens outcomes in Chronic Obstructive Pulmonary Disease (COPD), and reduced sleep quality is common in these patients. Thus, objective sleep monitoring is needed, but polysomnography (PSG) is cumbersome and costly. The WatchPAT determines sleep by a pre-programmed algorithm and has demonstrated moderate agreement with PSG in detecting sleep stages in normal subjects and in OSA patients. Here, we validated WatchPAT against PSG in COPD patients, hypothesizing agreement in line with previous OSA studies. 16 COPD patients (7 men, mean age 61 years), underwent simultaneous overnight recordings with PSG and WatchPAT. Accuracy in wake and sleep staging, and concordance regarding total sleep time (TST), sleep efficiency (SE), and apnea hypopnea index (AHI) was calculated. Compared to the best fit PSG score, WatchPAT obtained 93% sensitivity (WatchPAT = sleep when PSG = sleep), 52% specificity (WatchPAT = wake when PSG = wake), 86% positive and 71% negative predictive value, Cohen’s Kappa (κ) = 0.496. Overall agreement between WatchPat and PSG in detecting all sleep stages was 63%, κ = 0.418. The mean(standard deviation) differences in TST, SE and AHI was 25(61) minutes (p = 0.119), 5(15) % (p = 0.166), and 1(5) (p = 0.536), respectively. We conclude that in COPD-patients, WatchPAT detects sleep stages in moderate to fair agreement with PSG, and AHI correlates well.

## Introduction

Chronic obstructive pulmonary disease (COPD) is associated with major morbidity and mortality, with a global prevalence of more than 10%^1,2^. COPD patients report more difficulties in initiating and maintaining sleep than the general population, and more than 50% of COPD patients report excessive daytime sleepiness^3,4^. Obstructive sleep apnea (OSA) is another common disorder and public health care burden leading to excessive daytime sleepiness^5,6^. The prevalence of OSA in elderly COPD patients referred to pulmonary rehabilitation has been shown to be as high as 66% in one study, and at our hospital we previously found 75% of moderate to severely ill COPD patients having an apnea/hypopnea index (AHI) ≥ 5^7,8^. Patients with both COPD and OSA have greater morbidity and mortality compared to those with COPD or OSA alone^9^. Thus, diagnostic sleep monitoring is often needed in COPD.

Polysomnography (PSG) is considered the gold standard for evaluating sleep and diagnosing OSA. However, PSG has several disadvantages; it is cumbersome for the patients and both hook-up and manual sleep scoring is time consuming, making it expensive and limiting the availability. Therefore, unattended portable monitors for simple diagnosis of OSA were included in the 2007 American Academy of Sleep Medicine (AASM)^9^. Presently in Europe, only about 1/3 of the OSA diagnoses are established by the use of PSG, the rest mainly by respiratory polygraphy (PG)^10,11^. As COPD patients often have fragmented sleep, and because PG does not record whether the patient is actually sleeping, the AASM recommends PSG in evaluating sleep disordered breathing in COPD^9^.

Respiratory events are known to trigger surges of sympathetic activity in alpha-adrenergic receptors, resulting in attenuation of digital, peripheral arterial tone (PAT). Recordings of this PAT-signal, as well as arterial oxygen saturation by pulse oximetry (SpO2), heart rate, snoring and body movement is utilized by the WatchPAT, a portable device estimating not only respiratory events but also sleep/wake stages. Comparing WatchPAT with PSG in OSA patients, Pang *et al*. demonstrated a high correlation (r = 0.93) for AHI^12^. In a multi-center study of normal subjects and OSA patients, Hedner and colleagues found no significant differences in total sleep time (TST) and sleep efficiency (SE), and a moderate agreement (Cohen’s Kappa (κ) coefficient of 0.48) in detecting wakefulness, light sleep, deep sleep and rapid eye movement (REM) sleep^12–14^. COPD and the medication used by these patients often causes increased sympathetic activity^15,16^. As the PAT signal based on sympathetic activity is central in the WatchPAT sleep scoring and breath analysis algorithm, it is plausible that the accuracy of the WatchPAT in COPD patients may differ from that in OSA patients and the normal population.

In this study we present the results from an investigation where WatchPAT was used to estimate sleep quality and AHI in COPD patients. The WatchPAT results were compared to PSG recordings analysed by two technicians, blinded from each others’ results and from the automated WatchPAT score.

## Material and Methods

### Subjects

Patients attending a 4 week, in-patient pulmonary rehabilitation program at the LHL-clinics, Glittre, Norway, were recruited from September 2016 to June 2017. Prior to inclusion, a diagnosis of COPD in a stable state was verified by pulmonary function tests including post-bronchodilator spirometry, diffusion capacity of the lungs, body plethysmography and blood gas assessment. Exclusion criteria were COPD exacerbation within last 3 weeks, chronic respiratory failure (daytime arterial oxygen pressure (PaO2) ≤ 7.3 kPa or daytime arterial carbon dioxide pressure (PaCO2) ≥ 6.3 kPa), other diseases significantly affecting upper and lower airway function, a prior diagnosis of OSA, non-sinus cardiac arrhythmias, implanted pacemaker, coronary arterial disease with unstable angina pectoris or myocardial infarction last 3 months, uncontrolled hypertension, history of cerebral infarction, use of nitrate medication. All subjects used prescribed medication, but no respiratory depressant drugs were taken from 48 hours prior to first PSG-recording until end of study.

Informed consent was obtained from all individual participants included, all procedures performed were in accordance with the 1964 Helsinki declaration and its later amendments, and the study protocol was approved by The Regional Committee for Medical Research Ethics in Mid-Norway (2016/1360/rek midt).

### Study design

**Figure 1:**
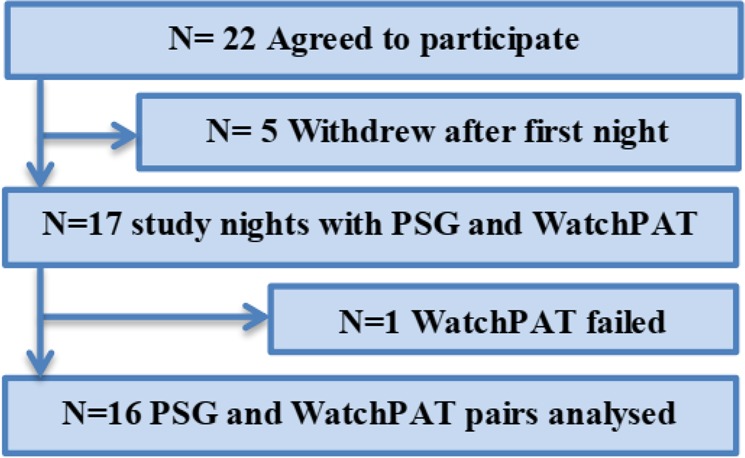
Selection of study population.

Double-blind, randomized, crossover, intervention trial in which patients consumed 70 mL of either beetroot juice containing nitrate or placebo immediately before bedtime. Subjects slept three nights semi-unattended in their hospital room, hooked up to PSG and WatchPAT simultaneously. The first night for acquaintance with the equipment (no recording was done), the second and third nights were block randomized to nitrate intervention or control (placebo) according to sex and age. The pre-programmed WatchPAT and the PSG recording were started when the patient went to bed, and stopped by the department nurse when the patient wanted to get up in the morning. The nurse reported on time for lights off. Here, data from the control nights are presented (Fig. 1), whereas results from the nitrate intervention are published elsewhere.

### Polysomnography

PSG was recorded by Embla A10 (Medcare Flaga, Reykjavik, Iceland) according to the 2007 recommendations from the AASM^17^, including electroencephalogram (F4-M1, C4-M1, O2-M1), left and right electrooculogram, and electromyogram submentalis monitoring. In addition, an electromyography of anterior tibialis, thoracic and abdominal breathing movements, nasal airflow pressure, SpO2, and body position were measured. All signals were sampled using Somnologica Studio Version 3.3 software (Medcare Flaga).

As PSG is manually scored it implies some inter-rater variability, which should be addressed when comparing agreement with other devices. Thus, the PSG’s were scored independently by two experienced registered polysomnography technicians (RPSGT’s) according to the rules set by the AASM^18^. An apnea was scored when the nasal pressure dropped ≥90% for ≥10  econds. A hypopnea was scored when the nasal pressure dropped ≥30% for ≥10 seconds, causing a ≥4% drop in SpO2 from baseline. Parameters used for analyses include stage score (Wake, N1, N2, N3, REM sleep) for each epoch as well as TST, sleep efficiency (SE, percentage of sleeping time between lights off and lights on) and AHI.

### Peripheral arterial tonometry

The WatchPAT (version 200, Itamar Medical, Israel) is a wrist worn sleep data recorder, connected to a finger probe with a plethysmograph and a pulse oximeter, and a microphone with an actigraph taped below the sternal notch. Recordings were analysed by the software zzzPAT (Itamar Medical, Israel). Stages (wakefulness, light sleep, deep sleep, REM sleep) were scored in 30-second epochs based on spectral components from the PAT-signal. Respiratory events (apneas, hypopneas) were scored based on PAT-signal amplitude, heart rate and SpO2. The algorithm was set to score hypopneas in the presence of ≥4% drop in SpO2. Parameters used for analyses include stage score (wakefulness, light sleep, deep sleep, REM sleep) for each epoch as well as TST, SE and AHI.

### Outcomes and measurements

Prior to the epoch-by-epoch comparisons, the temporal synchronization between WatchPAT and PSG recordings were verified using the internal clock, position and movement recordings from both devices.

First, we analysed WatchPAT’s agreement with the RPSGT’s in scoring sleep and wake by calculating sensitivity (e.g. WatchPAT = sleep when PSG = sleep) and specificity (e.g. WatchPAT = wake when PSG = wake). Then we calculated WatchPAT’s positive and negative predictive values, considering the PSG’s as gold standard. Subsequently, we performed an epoch-by-epoch comparison of sleep/wake stages, the PSG-scored stages N1 and N2 considered equal to WatchPAT’s light sleep. Finally, the agreement in terms of TST, SE and AHI were calculated. All the above mentioned outcomes were also analysed comparing the results from the two RPSGT’s.

### Statistical analysis

In their OSA study comparing WatchPAT to PSG, Pang *et al*. found AHI correlating with Pearson’s *r* = 0.929, 95% confidence interval 0.858-0.965^12^. Assuming we would find an equivalent correlation in COPD patients, and a 5% level of significance with 80% power, a sample size of 15 subjects was calculated a priori.

Agreement was considered when an epoch had the same stage by both scorers (WatchPAT or RPSGT), reported as percentage of agreement and as Cohen’s *κ* coefficients. Other correlations were calculated as Pearson’s *r*. Intra-class correlation coefficients (ICC) was calculated for TST, SE and AHI, and the mean differences in TST, SE and AHI were compared by one sample T-tests, inspection of Bland Altman plots and linear regression of the differences. Two-sided *P* values of ≤0,05 were considered significant. All analyses were performed using IBM SPSS Statistics version 25.

## Results

As shown in Fig. 1, five subjects withdrew after the first night, main reason was fear of not being able to sleep with electrodes on head and body. One sleep study was excluded as the WatchPAT did not start recording.

Thus, results from 16 patients were analyzed (7 men, two current smokers), with a mean (standard deviation) age 61.4(9.1) years, body mass index (BMI) 26.4(5.3), post-bronchodilator forced expiratory volume in one second (FEV1) 46.1(19.6) % of expected, residual volume/total lung ratio volume (RV/TLC) 0.56(0.12), PaO2 9.1(1.0) kPa, PaCO2 5.0(0.6) kPa, Epworth sleepiness scale score 5.3(2.5).

### Sleep wake status

WatchPAT agreements with polysomnography technician number one (RPSGT#1) and two (RPSGT#2) in scoring sleep (specificity) were 93.4% and 93.6%, whereas agreements in wake (sensitivity) were 51.5% and 50.7%. Cohen’s κ for detecting sleep from wake was 0.496 and 0.492. The inter-scorer agreements between RPSGT#1 and RPSGT#2 was 97.9% in scoring sleep, and 96.0% for wake (κ 0.931).

WatchPAT’s ability to detect sleep (positive predictive value) was 85.9% and 85.4%, whereas it correctly scored wake (negative predictive value) in 71.2% and 71.9% of the epochs. WatchPAT correctly predicted non rapid eye movement (NREM) sleep in 80.6% and in 79.8% of the epochs. Other positive predictive values are listed in Table 1.

**Table 1:** WatchPAT’s positive predictive values in stages wake, light sleep (N1 + N2), deep sleep (N3) and REM sleep.

	WP vs RPSGT#1	WP vs RPSGT#2	RPSGT#2 vs RPSGT#1
Wake	71.2	71.9	96.0
Light sleep	67.9	64.4	88.8
Deep sleep	29.6	36.8	85.3
REM sleep	62.2	59.7	92.7

Percentage of WatchPAT scored epochs where agreement exists with PSG out of the total number of WatchPAT scored epochs in the specified stage.

WP = WatchPAT, RPSGT#1 and RPSGT#2 = polysomnographies scored by polysomnography technician number one and two, respectively. N1 + N2 = non rapid eye movement sleep (NREM) stage one and two, N3 = NREM stage 3, REM = rapid eye movement.

### Overall agreement

When the WatchPAT results were compared epoch-by-epoch to the PSG scorings, overall agreement with RPSGT#1 in wake, light sleep (N1 + N2), deep sleep (N3) and REM sleep was found in 9279 out of 14670 epochs, and with RPSGT#2 in 9061 out of 14650 epochs (63.3% and 61.8%, Cohen’s *κ* 0.418 and 0.407, respectively). RPSGT#2 agreed with RPSGT#1 in 13382 out of 14751 epochs (90.7%, *κ* 0.856). There was considerable inter-individual differences between the study subjects (Table 2). However, age, gender, COPD severity (as characterised by FEV1, RV/TLC ratio, BMI, PaO2, PaCO2) and daytime sleepiness (Epworth score) showed no significant correlations with the percentage of sleep score agreement or Cohen’s κ. In subject #1 and #2, a total of 948 epochs were scored as wake by RPSGT#1, but only 274(28.9%) of these were scored as wake by WatchPAT, the rest as light sleep 526(55.5%), deep sleep 59(6.2%) and REM sleep 89(9.4%). Equivalent numbers were found between WatchPAT and RPSGT#2.

**Table 2:** Epoch-by-epoch overall agreements in the detection of wake, light sleep, deep sleep and REM sleep, as percent and as Cohen’ κ coefficient.

Subject #	RPSGT#1 vs WP			RPSGT#2 vs WP			RPSGT#2 vs RPSGT#1		
	%	κ	N	%	κ	N	%	κ	N
1	36.2	0.087	916	36.8	0,087	915	92.3	0.880	924
2	47.2	0.215	927	47.0	0.216	925	97.0	0.939	925
3	62.6	0.473	846	60.7	0.418	845	89.1	0.824	845
4	59.3	0.369	760	60.0	0.384	758	93.5	0.887	758
5	70.6	0.510	856	70.9	0.513	855	95.6	0.927	855
6	79.3	0.542	911	66.3	0.382	909	82.0	0.686	909
7	79.2	0,660	1018	78.7	0.657	1017	93.7	0.898	1020
8	71.8	0.387	909	72.0	0.395	908	92.6	0.863	990
9	54.3	0.301	957	54.4	0.310	956	92.9	0.882	956
10	62.2	0.460	793	61.6	0.468	792	91.7	0.886	793
11	68.6	0.528	789	56.8	0.498	787	87.0	0.789	787
13	63.3	0.332	915	64.5	0.376	913	87.6	0.757	913
15	59.9	0.342	972	57.0	0.316	971	88.6	0.833	971
19	64.3	0.438	928	67.9	0.504	927	91.2	0.848	927
21	66.8	0.472	1080	63.4	0.443	1080	90.1	0.848	1086
22	64.0	0.466	1093	61.6	0.440	1092	87.2	0.811	1092
All subjects	63.3%	0.418	14670	61.8%	0.407	14650	90.7%	0.856	14751

The overall agreement was defined as the number of epochs where agreement exists in any specific state divided by the total number of epochs. WP = WatchPAT, RPSGT#1 and RPSGT#2 = polysomnographies scored by registered polysomnography technician number one and two, respectively. N = number of epoch pairs.

### Total sleep time, sleep efficiency and apnea hypopnea index

As shown in Table 3, ICC coefficients for WatchPAT versus each of the RPSGT’s regarding TST were low, but Table 4 shows that no significant difference in TST was found. Notably, the 95% confidence intervals for the ICC standard deviations were high, and the 95% confidence intervals for TST difference ranged from −7 to +57 minutes (WatchPAT versus RPSGT#1) and from −3 to 59 minutes (WatchPAT versus RPSGT#2). Likewise, for the difference in sleep efficiency the 95% confidence intervals were −2% to +13% between WatchPAT and each of the RPSGT’s.

**Table 3:** Intra-class correlation coefficients (95% confidence interval) for total sleep time, sleep efficiency and apnea/hypopnea index.

	WP versus RPSGT#1	p	WP versus RPSGT#2	p	RPSGT#2 versus RPSGT#1	p
TST	0.488(−0.311–0.814)	0.087	0.497(−0.254–0.815)	0.074	0.997(0.991–0.999)	*0.000*
SE	0.375(−0.626–0.774)	0.174	0.414(−0.487–0.786)	0.138	0.997(0.993–0.999)	*0.000*
AHI	0.957(0.878–0.985)	*0.000*	0.954(0.872–0.984)	*0.000*	0.997(0.993–0.999)	*0.000*

N = 16. TST total sleep time, SE = sleep efficiency, AHI = apnea/hypopnea index, WP = WatchPAT, RPSGT#1 and RPSGT#2 = registered polysomnography technician number one and two, respectively.

**Table 4:** Mean value (standard deviation) and mean difference in Total Sleep Time, Sleep Efficiency and Apnea Hypopnea Index as scored by WatchPAT, RPSGT#1 and RPSGT#2.

	WP	RPSGT#1	RPSGT#2	WP- RPSGT#1	p	WP- RPSGT#2	p	RPSGT#2 - RPSGT#1	p
TST	376 (39)	351 (64)	348(62)	25 (61)	0.119	28(58)	0.075	−2.9(6.5)	0.097
SE	82.8(7.7)	76.5(14.7)	75.4(14.2)	5.3(14.6)	0.166	5.8(14.1)	0.124	−0.4(1.4)	0.243
AHI	9.6(11.9)	8.8(12.5)	8.4(13.3)	0.8(5.1)	0.536	1.2(5.3)	0.372	−0.4(1.3)	0.209

N = 16. TST = minutes total sleep time, SE = percentage sleep efficiency, AHI = number of apneas and hypopneas per hour of sleep. WP = WatchPAT, RPSGT#1 and RPSGT#2 = polysomnographies scored by registered polysomnography technician number one and two, respectively.

Inspection of Bland Altman plots showed that subject #2 differed highly between WatchPAT and the two RPSGT’s, both in TST (188 and 187 min’s) and SE (40.6% and 40.3%). However the AHI-differences in this subject were only 3.5 and 3.1, and neither WatchPAT nor the RPSGT’s reported AHI ≥ 5 (i.e. not OSA). Linear regression did not show any proportional bias in the distribution of the TST and SE differences when controlling for subject#2.

The WatchPAT–PSG differences in TST were positively correlated with Cohen’s κ for detecting all sleep stages (RPSGT#1: *r* = 0.601, p = 0.014; and RPSGT#2: *r* = 0.628, p = 0.009). Positive correlations of similar magnitude were also found for SE differences vs. Cohen’s κ, as well as for TST differences and SE differences vs. percentage of wake and sleep stage agreement. In other words; when WatchPAT failed in sleep staging, TST and SE was affected.

**Figure 2:**
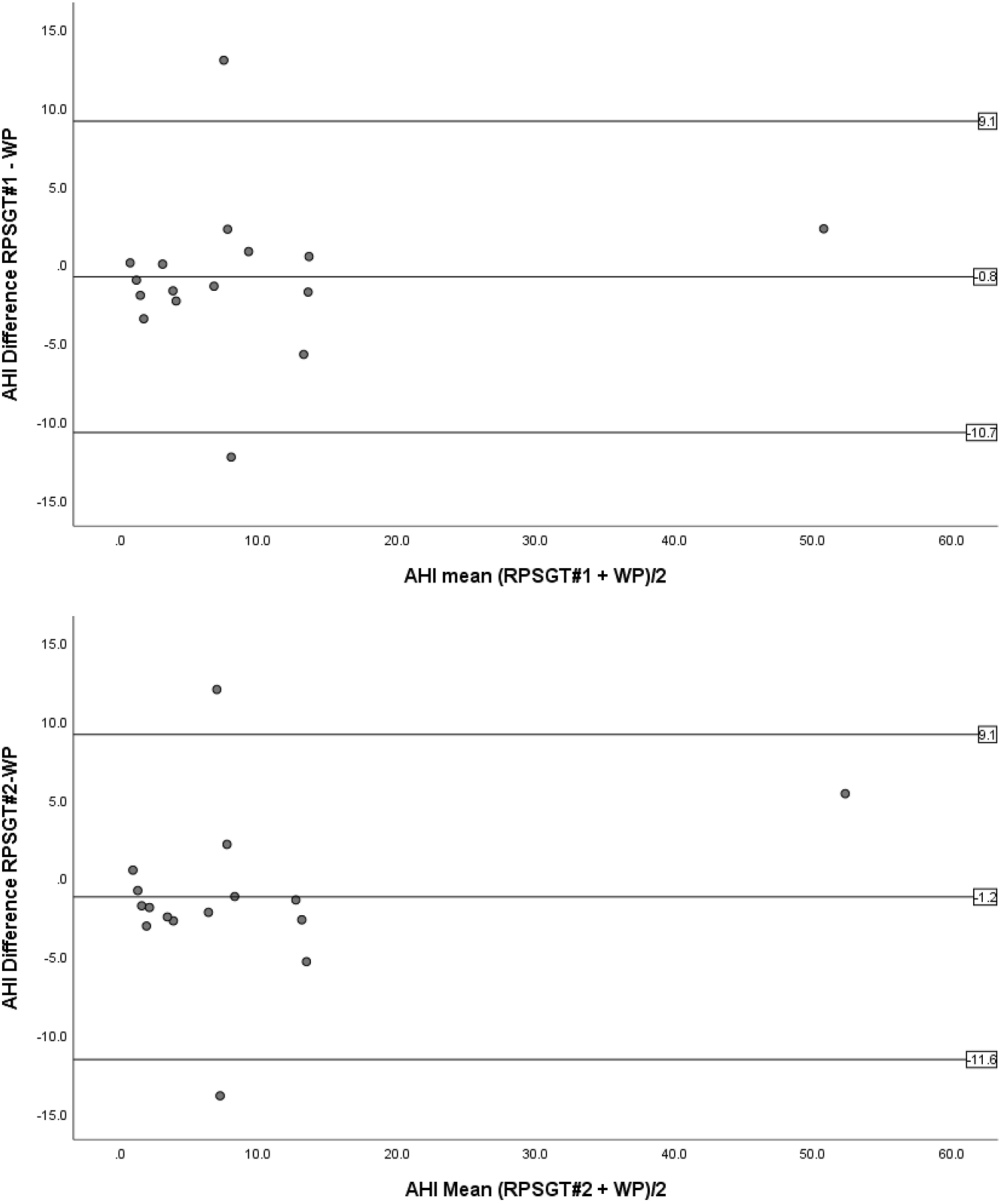
Bland-Altman Plots of apnea-hypopnea index. Horizontal lines represent mean difference (middle line) and 95% confidence interval (upper and lower lines). AHI = apnea-hypopnea index, RPSGT#1 and RPSGT#2 = polysomnography technician number one and number two, respectively.

Intra-class correlation coefficients for AHI were high as shown in Table 3, and the WatchPAT’s AHI did not differ significantly from the RPSGT’s on a group level (Table 4). However, as indicated in Fig. 2, WatchPAT failed to detect correct OSA severity in two patients. In patient #19, AHI scored by WatchPAT was 1.0 whereas the RPSGT’s scored 14.0 and 13.0 (TST 356 min’s, 410 min’s and 402 min’s, respectively), and in patient #22, WatchPAT scored 14.2 vs 1.9 and 0.3 (TST 401 min’s, 381 min’s and 382 min’s) scored by RPSGT#1 and RPSGT #2, respectively. All subjects were scored likewise according to OSA severity by both RPSGT’s (Table 5).

**Table 5:** Number of subjects in each category of severity according to apneas and hypopneas per hour of sleep.

	Normal (AHI < 5)	Mild AHI 5–14.9	Moderate AHI 15–29.9	Severe AHI ≥ 30
WatchPAT	7	7	1	1
RPSGT#1	8	7	0	1
RPSGT#2	8	7	0	1

RPSGT#1 and RPSGT#2 = polysomnographies scored by registered polysomnography technician number one and two, respectively. AHI = number of apneas and hypopneas per hour of sleep.

## Discussion

This study of patients with moderate to severe COPD demonstrated that the WatchPAT scores sleep with less agreement with PSG compared to previous studies of healthy individuals and OSA patients. TST and SE estimated by WatchPAT does not correlate with PSG, however, despite wide individual variability in TST and SE the differences are not statistically different on a group level. The WatchPAT’s AHI is reasonably accurate.

Although we found the automated WatchPAT’s sensitivity in detecting sleep to be good, it’s specificity (as scoring wake when the RPSGT’s scored wake) was only about 51%; compared to the 66–74% found by Hedner *et al*. in non-COPD subjects^19^. We found WatchPAT’s abilities in predicting wake, REM and light sleep to be in line with Hedner’s findings, however, it correctly scored deep sleep in only 30–37% of the epochs, compared to 69% found by Hedner^14^. In two subjects, more than half of the epochs scored as wake by the RPSGT’s were scored as light sleep by WatchPAT. As TST and SE were overestimated in these subjects and underestimated in others, the mean values did not differ significantly between the WatchPAT and the PSG recordings, but the individual differences could not be explained by demographic or COPD severity indices. Interestingly, although the TST difference between WatchPAT and the RPSGT’s in subject #2 was more than 3 hours, the AHI differed only between 3.1 and 3.5, and the AHI was scored <5 both by WatchPAT and the RPSGT’s. Thus, differences in sleep status did not seem to affect the AHI in this case, but in subjects #19 and #22, significant AHI differences of >10 was accompanied by TST differences of approx. 50 min’s and 20 min’s, respectively.

TST and SE were moderately correlated with the wake/sleep agreement, both according to percentage and as Cohen’s κ. The WatchPAT’s epoch-by-epoch agreement with the RPSGT’s in scoring wake, light sleep, deep sleep and REM sleep varied between the study subjects from 36% to 79%, with Cohen’s κ varying from 0.09 to 0.66 (Table 2).

Jacob Cohen suggested the κ result to be interpreted as fair in the interval 0.21–0.40 and moderate in the interval 0.41–0.60^20^. Thus, the overall agreement between WatchPAT and the RPSGT’s with a κ of 0.41–0.42 can be described as fair to moderate, in contrast to the κ of 0.48 previously found in normal subjects and OSA-patients published by Hedner’s group^14^. However, no definite consensus is established in considering what is an adequate agreement, some statisticians describe κ in the range 0.40–0.59 as weak, arguing that little confidence should be placed in results with κ < 0.60^21^.

The association between autonomic output and sleep states has previously been extensively studied, showing different autonomic patterns in wake, NREM and REM sleep as reflected in heart rate, heart rate variability, blood pressure and combinations of these^22–25^. In the transition from wake to sleep, sympathetic activity decreases and parasympathetic activity increases, with further progress as sleep is consolidated. REM sleep, however, is associated with considerable sympathetic variability and smaller changes in parasympathetic activity, resulting in profound changes in vasoconstriction and heart rate variability in the PAT-recordings^25,26^. In OSA, peak sympathetic activity is associated with the termination of respiratory events^27^. The algorithms in WatchPAT for detecting sleep/wake status and differentiating between light sleep, deep sleep and REM-sleep, as well as identifying respiratory events (e.g. apneas and hypopneas) are based on these associations. In sleeping COPD patients, a marked worsening of breathing and arterial blood gases are often seen, especially during REM-sleep, in contrast to healthy individuals where sleep usually represent a restful period for the pulmonary and cardiovascular systems^7,28^. Arterial stiffness is increased in sleeping COPD patients compared to healthy controls, particularly during REM sleep, and their vascular tone was reduced in response to nasal high flow oxygen therapy^29,30^. It is unclear if the WatchPAT algorithm can identify and correct for these COPD specific changes in the PAT-signal, blood gases and heart rate variability. Thus, it is possible that the low κ correlation between WatchPAT and the PSG scores (approximately half of the study subjects had κ < 0.40) is due to autonomic dysfunction in some of the COPD patients. Likewise, the WatchPAT-PSG differences in AHI found in study subjects #19 and #22 can be due to changed sympathetic activity from altered lung mechanics, hypoxia and/or hypercapnia. Finally, Hedner’s group found that greater movement intensity in patients with severe OSA was associated with less agreement in sleep/wake status between the two methods, and pointed out that overestimation of sleep time appears to be a general limitation of actigraphy^19^. Thus, the low specificity and the wide individual differences we found in sleep staging, TST and SE might not depend on the chronic obstructive lung disease *per se*, but rather on the sleeping patients’ movements. On the other hand, despite the fact that half of the study population had a RPSGT-scored AHI ≥ 5 (Table 5), only one of these had AHI ≥ 15, indicating that the severity of Overlap COPD-OSA in this population was low.

To our knowledge, this is the first study comparing WatchPAT with PSG in COPD patients. As such, it has some limitations. First, the sample size was calculated on the assumption that the correlation would be in line with previous studies of OSA-patients. We fount the best match ICC coefficient (95% confidence interval) for AHI to be 0.96 (0.88–0.99), indeed comparable to Pang *et al*.'s Pearson’s *r* of 0.93 (0.86–0.97)^12^, but with only sixteen study subject, identifying possible characteristics in COPD that mislead the WatchPAT algorithm was not possible.

Also, although we synchronized the WatchPAT and PSG epochs by the internal clock, position and movement signals, an off-set of up to 30 seconds is possible. This implies that the first and last epoch in a given sleep stage may not match, resulting in several mismatches in very fragmented sleep. However, with more than 14600 epochs compared in all, we find it unlikely that this would significantly affect the agreement between the two methods at a group level.

COPD patients can have other sleep related breathing disorders than OSA, e.g. sleep related hypoventilation (SH)^7^, and as we did not record PCO2 during sleep it is possible that SH can explain some of the differences between WatchPAT and the PSG scorings.

Finally, the zzzPAT does not report apneas and hypopneas on a continuous timeline, only as the total number of events, and in this study the RPSGT’s did not score hypopneas as obstructive or central. Thus, we were not able to explore the major differences in AHI found in two of the subjects.

## Conclusion

We have compared automated sleep scoring by WatchPAT to the gold standard PSG in moderate to severely ill COPD patients in a stable state of their disease, and found a good sensitivity (WatchPAT = sleep when PSG = sleep) but a weak specificity (WatchPAT = wake when PSG = wake). The WatchPAT underestimates deep NREM sleep and shows only fair to moderate agreement with PSG in the overall detection of wake, NREM light sleep, NREM deep sleep and REM sleep. TST and SE does not differ significantly, but wide individual differences makes WatchPAT’s usefulness in evaluating sleep quality questionable in these patients. However, AHI shows reasonably good correlation.

Autonome dysfunction in COPD can be a possible explanation for the discrepancies found here as compared to previous studies in normal populations and in OSA-patients, but our study was too small to explore such associations.

Until proven otherwise in bigger studies, we conclude that the WatchPAT must be used with caution for sleep evaluation in COPD, but AHI correlates well enough for screening of OSA in these patients.

## Data Availability

The datasets generated and analysed during this study are available from the corresponding author on reasonable request.

## References

[1] Lopez A. D. (2006). Chronic obstructive pulmonary disease: current burden and future projections. European Respiratory Journal.

[2] Adeloye D (2015). Global and regional estimates of COPD prevalence: Systematic review and meta-analysis. J Glob Health.

[3] Cormick W, Olson LG, Hensley MJ, Saunders NA (1986). Nocturnal hypoxaemia and quality of sleep in patients with chronic obstructive lung disease. Thorax.

[4] Klink, M. & Quan, S. F. Prevalence of reported sleep disturbances in a general adult population and their relationship to obstructive airways diseases. *Chest*, **91**, 540–546 (1987/4). 10.1378/chest.91.4.540 3829746

[5] Peppard PE (2013). Increased prevalence of sleep-disordered breathing in adults. Am. J. Epidemiol..

[6] Leger D, Bayon V, Laaban JP, Philip P (2012). Impact of sleep apnea on economics. Sleep medicine reviews.

[7] Holmedahl NH, Overland B, Fondenes O, Ellingsen I, Hardie JA (2014). Sleep hypoventilation and daytime hypercapnia in stable chronic obstructive pulmonary disease. Int J Chron Obstruct Pulmon Dis.

[8] Soler X (2015). High Prevalence of Obstructive Sleep Apnea in Patients with Moderate to Severe Chronic Obstructive Pulmonary Disease. Ann Am Thorac Soc.

[9] Owens, R. L. & Malhotra, A. Sleep-disordered breathing and COPD: the overlap syndrome. *Respir. Care* **55**, 1333–1344; discussion 1344–1336 (2010). PMC3387564 20875160

[10] Collop NA (2007). Clinical guidelines for the use of unattended portable monitors in the diagnosis of obstructive sleep apnea in adult patients. Portable Monitoring Task Force of the American Academy of Sleep Medicine. Journal of clinical sleep medicine: JCSM: official publication of the American Academy of Sleep Medicine.

[11] Hedner J (2011). The European Sleep Apnoea Database (ESADA): report from 22 European sleep laboratories. Eur. Respir. J..

[12] Pang KP, Gourin CG, Terris DJ (2007). A comparison of polysomnography and the WatchPAT in the diagnosis of obstructive sleep apnea. Otolaryngol. Head Neck Surg..

[13] Zou D, Grote L, Peker Y, Lindblad U, Hedner J (2006). Validation a portable monitoring device for sleep apnea diagnosis in a population based cohort using synchronized home polysomnography. Sleep.

[14] Hedner J (2011). Sleep staging based on autonomic signals: a multi-center validation study. Journal of clinical sleep medicine: JCSM: official publication of the American Academy of Sleep Medicine.

[15] Newton GE, Azevedo ER, Parker JD (1999). Inotropic and sympathetic responses to the intracoronary infusion of a beta2-receptor agonist: a human *in vivo* study. Circulation.

[16] van Gestel AJ, Steier J (2010). Autonomic dysfunction in patients with chronic obstructive pulmonary disease (COPD). Journal of thoracic disease.

[17] Iber C, Ancoli-Israel S, Chesson AL & Quan SF, e. *The AASM manual for the scoring of sleep and associated events*. *Rules, terminology and technical specifications*. (American academy of sleep medicine 2007).

[18] Berry RB (2012). Rules for scoring respiratory events in sleep: update of the 2007 AASM Manual for the Scoring of Sleep and Associated Events. Deliberations of the Sleep Apnea Definitions Task Force of the American Academy of Sleep Medicine. Journal of clinical sleep medicine: JCSM: official publication of the American Academy of Sleep Medicine.

[19] Hedner J (2004). A novel adaptive wrist actigraphy algorithm for sleep-wake assessment in sleep apnea patients. Sleep.

[20] Cohen J (1960). A Coefficient of Agreement for Nominal Scales. Educational and Psychological Measurement.

[21] McHugh ML (2012). Interrater reliability: the kappa statistic. Biochem Med (Zagreb).

[22] Negoescu RM, Csiki IE (1989). Autonomic control of the heart in some vagal maneuvers and normal sleep. Physiologie.

[23] Penzel T (2000). Heart rate variability during sleep stages in normals and in patients with sleep apnea. Stud. Health Technol. Inform..

[24] Trinder J (2001). Autonomic activity during human sleep as a function of time and sleep stage. J. Sleep Res..

[25] Shinar Z, Akselrod S, Dagan Y, Baharav A (2006). Autonomic changes during wake-sleep transition: a heart rate variability based approach. Auton Neurosci.

[26] Lavie P, Schnall RP, Sheffy J, Shlitner A (2000). Peripheral vasoconstriction during REM sleep detected by a new plethysmographic method. Nat. Med..

[27] Hedner J, Ejnell H, Sellgren J, Hedner T, Wallin G (1988). Is high and fluctuating muscle nerve sympathetic activity in the sleep apnoea syndrome of pathogenetic importance for the development of hypertension?. J. Hypertens. Suppl..

[28] Flenley DC (1985). Sleep in chronic obstructive lung disease. Clin. Chest Med..

[29] Grote L (2017). REM Sleep Imposes a Vascular Load in COPD Patients Independent of Sleep Apnea. Copd.

[30] Fricke K (2018). Nasal high flow, but not supplemental O2, reduces peripheral vascular sympathetic activity during sleep in COPD patients. Int J Chron Obstruct Pulmon Dis.

